# Comparison of the effectiveness and safety of unilateral and bilateral percutaneous vertebroplasty for osteoporotic vertebral compression fractures: A protocol for systematic review and meta-analysis

**DOI:** 10.1097/MD.0000000000028453

**Published:** 2021-12-23

**Authors:** Yu Chen, Huang Zhang, Huihong Chen, Zhiliang Ou, Yiping Fu, Jinjun Zhang

**Affiliations:** The Third Department of Surgery, Xianyou County Hospital, Fujian, China.

**Keywords:** bilateral puncture, meta-analysis, percutaneous vertebral plastic, unilateral puncture

## Abstract

**Background:**

The objective of this study was to compare the efficacy of lateral and bilateral percutaneous vertebroplasty (PVP) in treating osteoporotic vertebral compression fractures (OVCFs).

**Methods:**

A comprehensive literature search was performed using PubMed, Cochrane Library, EMBASE, CMB, CNKI, Wanfang, and VIP databases between January 2014 and December 2020. The clinical efficacy of the 2 approaches was evaluated by comparing perioperative outcomes (operation time, X-ray exposure time, volume of injected cement), clinical outcomes (degree of vertebral height restoration, improvement of Cobb angle, visual analogue scale score, and Oswestry Disability Index scores), and operation-related complications (rate of cement leakage, adjacent vertebral fracture rate, and nerve root stimulation). Data were analyzed using RevMan 5.3.3 and Stata 15.1.

**Results:**

A total of 237 related articles were retrieved, and 17 randomized controlled trials were included. Meta-analysis results showed that compared to bilateral PVP, unilateral PVP led to decreased operation times (mean difference [MD] = −15.24, 95% confidence interval [CI]: [−17.77, −12.70], *P* < .05), decreased X-ray exposure time (MD-8.94, 95% CI[−12.08,−5.80]; *P* < .01), decreased volumes of injected cement (MD-1.57, 95% CI[−2.00,−1.14]; *P* < .05), and lower incidence of cement leakage (risk ratio [RR] = 0.6,95% CL[0.48,0.77], *P* < .01). Patients that underwent unilateral PVP experienced more effective pain relief at the last follow-up (MD-0.09, 95% CI [−0.15,−0.03];*P*=.006 < .05) and had a low degree of vertebral height restoration (MD-0.38, 95% CL [−0.71, −0.06]; *P*=.02 < .05). However, no differences in adjacent vertebral fractures (RR 1.19, 95% CI [0.78,1.82]; *P* = .41 > .01), nerve root stimulation (RR 1.98, 95% CI [0.22, 17.90]; *P* = .54 > .01), improvement of Cobb angle (MD = −0.18, 95% CI [−0.49, 0.13], *P* = .26 > .01), and improvement of ODI score (MD = 0.22, 95% CI[−0.37, 0.80], *P* > .05) were found between the 2 approaches.

**Conclusions:**

Although both unilateral and bilateral PVP can improve the quality of life of this patient population by managing pain effectively, unilateral PVP offers more benefits, including shorter operation time and less fluoroscopy, and should be recommended in clinical practice for OVCFs.

## 1 Introduction

Due to a growing aging population, the annual incidence rate of osteoporotic vertebral compression fractures (OVCFs) in China has increased substantially over the past few years. OVCFs can cause chronic back pain and limit elderly mobility, along with fatigue, depression, and insomnia.^[1]^ Current treatment options for OVCFs mainly include conservative and surgical options; however, conservative treatment cannot correct spinal deformities, and lumbago often lasts for a long time.^[1]^ As one of the most successful and effective minimally invasive treatment techniques for OVCFs, percutaneous vertebroplasty (PVP) has been widely used to treat elderly thoracolumbar osteoporotic fractures and has achieved satisfactory clinical results.^[2]^

PVP for OVCFs can be divided into unilateral and bilateral approaches; however, there is still a lack of consensus on the clinical safety and effectiveness of these 2 approaches. Two recent studies based on meta-analysis^[3,4]^ revealed that unilateral and bilateral punctures could efficiently relieve patient pain and improve quality of life; nonetheless, the unilateral puncture required shorter operation time, less fluoroscopy frequency, and bone cement injection volume. New comparative studies of these 2 PVP approaches have been published. Therefore, we searched the published data in various online databases and performed a meta-analysis to systemically compare which approach is safer and more effective.

## 2 Materials and methods

Ethical approval was not necessary because this is a meta- analysis. All data were available on the internet.

### 2.1 Search strategy

A comprehensive literature search was performed using the PubMed, Cochrane Library, EMBASE, CMB, CNKI, Wanfang database, and Weipu database from January 2014 to December 2020 according to the Method Guideline for Systematic Reviews published by the Cochrane Back Review Group editorial board. Keywords used to identify relevant studies were “osteoporotic,” “osteoporosis,” “vertebral compression fracture,” “vertebroplasty,” “unilateral,” “unipedicular,” “bilateral,” and “bipedicular.” MeSH terms were used, including “osteoporosis,” “compression fractures,” “osteoporotic fractures,” “spinal fractures,” and “vertebroplasty.”

### 2.2 Inclusion and exclusion criteria

The inclusion criteria consisted of:

1. Adult patients diagnosed with OVCFs
2. Interventional studies (RCTs)
3. Studies reported the comparisons between unilateral and bilateral PVP.
4. Studies reported the following outcomes: operation time, fluoroscopy, bone cement injection volume, vertebral body height, Cobb angle, cement leakage rate, adjacent vertebral fracture rate, nerve root stimulation, visual analogue scale (VAS), Oswestry Disability Index (ODI).

The exclusion criteria consisted of:

1. Pathological fractures caused by tumors;
2. Patients treated with kyphoplasty;
3. Review article, comments, meta-analysis, studies without related outcomes, and studies without RCT design.

### 2.3 Literature selection and quality evaluation

For our double blinded-study, the titles, abstracts and full text were screened and data extracted by 2 independent reviewers. Points of disagreement were reconciled or arbitrated by a third reviewer. The 2 reviewers evaluated the quality of the literature, using the Cochrane risk of bias tool, and the included literature was evaluated according to the following seven indicators: random sequence generation, allocation concealment, blinding of participants and personnel, blinding of outcome assessment, incomplete outcome data, selective reporting, and other bias. Each criterion was divided according to “yes,” “no” and “unclear,”“yes” indicated a low risk of bias, “no” indicated a high risk of bias, and “unclear” indicated an uncertain risk of bias.

### 2.4 Statistical methods

RevMan Manager Software (Version 5.3.3; Copenhagen: The Nordic Cochrane Centre, The Cochrane Collaboration, 2014) and Stata Statistical Software (Version 15.1; StataCorp 4905 Lakeway Dr College Station, TX 77845) were used to analyze the metadata, and *I*^2^ was used to test the heterogeneity between studies. If *I*^2^ < 50% and *P* > .1, it was suggested that the heterogeneity was small, and meta-analysis should be carried out using a fixed-effect model. *I*^2^ > 50% and *P* < .1 suggested significant heterogeneity, and subgroup analyses should be conducted to explore possible explanations for heterogeneity. The risk ratio (RR) was calculated as the effect size of binary variables, and the mean difference (MD) was calculated as the effect of continuous variables. Publication bias was investigated visually by inspecting funnel plots, and Begg and Egger tests were used to quantify the bias captured in the funnel plot (*P* < .05).

## 3 Results

### 3.1 The literature incorporates the results

**Figure 1:**
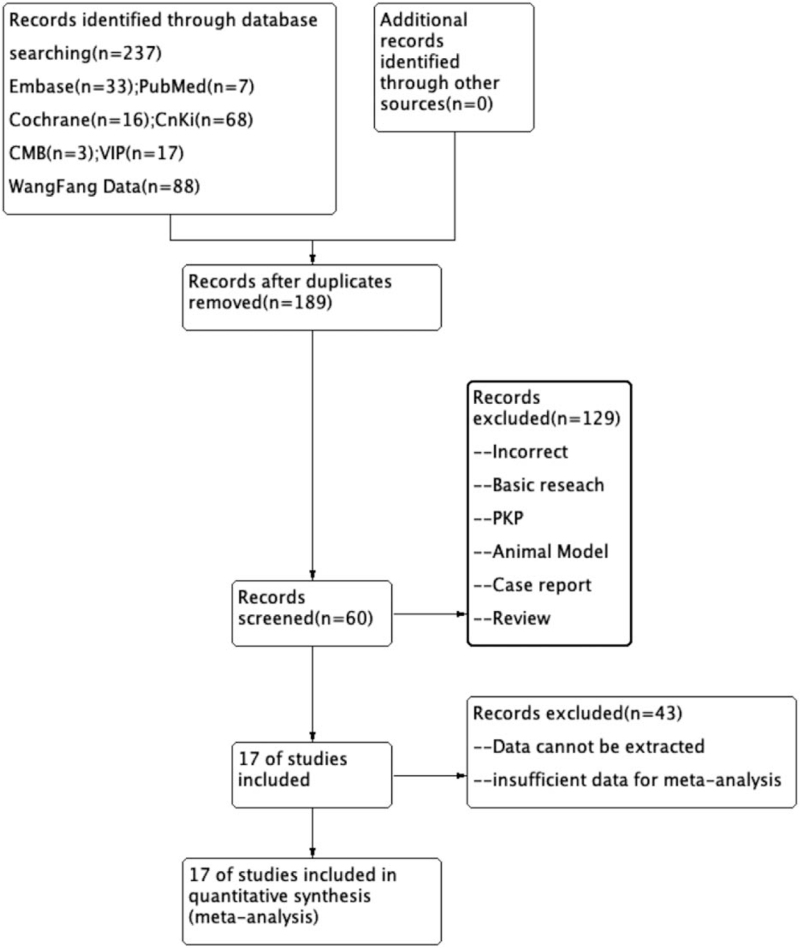
Flow diagram of the study selection.

In the initial literature search, 237 relevant articles were identified between January 2014 and December 2020. After removing duplicate studies, browsing the titles, and screening full texts, 17 studies^[5–21]^ were finally selected. A flow chart of the literature selection process is shown in Figure 1, and the basic characteristics of the included studies are shown in Table 1.

**Table 1:** Data of Included studies.

Characteristics of studies included in the meta-analysis.	Number of patients		Vertebral bodies		
	Unilateral	Bilateral	Unilateral	Bilateral	Outcome data
Lu CH, 2015^[5]^	297	252	347	296	T, V, O, C, S, F
Wang YF, 2018^[6]^	30	30	30	30	T, V, O, R, C, G
Zhou R, 2015^[7]^	41	38	41	38	T, R, G, S
Tang XS, 2019^[8]^	80	80	80	80	T, V, R, C, Z, G, S
Liu YH, 2018^[9]^	48	47	59	57	T, V, O, G, S
Wang Y, 2016^[10]^	30	30	30	30	T, R, C, Z, G, S, J
Zhou RH, 2016^[11]^	32	30	32	30	T, R, G, S
He XH, 2019^[12]^	43	45	43	45	T, V, O, C, Z, G, S
Fu JH, 2017^[13]^	45	45	45	45	T, V, O, R, C, S
Liu CL, 2015^[14]^	48	50	48	50	T, C, S, F
Wang LF, 2018^[15]^	34	34	34	34	T, O, C, Z
Wang WT, 2018^[16]^	151	140	151	140	T, V, O, R, G, S, J, F
Sun HB, 2019^[17]^	29	28	20	28	T, V, O, R, G, S
Zhang LG, 2015^[18]^	36	32	36	32	V, R, S
Zhang L, 2015^[19]^	24	26	24	26	T, V, O, R, G, S
Chen CM, 2014^[20]^	23	21	20	19	T, V, O, G, S
Cheng YH, 2019^[21]^	30	32	26	22	T, V, R, G, S
Total	1013	1082	947	1014	

C = Cobb angle, F = refracture rate, G = bone cement injection, J = nerve root stimulation, O = ODI, R = X-ray exposure, S = bone cement leakage rate, T = surgical time, V = VAS score, Z = vertebral height.

### 3.2 The basic characteristics of the included study

Seventeen studies were included in this meta-analysis, including a total of 1961 patients with 2096 spinal segments. A total of 1013 patients with 1082 segments were treated with unilateral PVP, and 947 patients with 1014 segments were treated with bilateral PVP (Table 1).

### 3.3 Included literature methodological assessment

**Figure 2:**
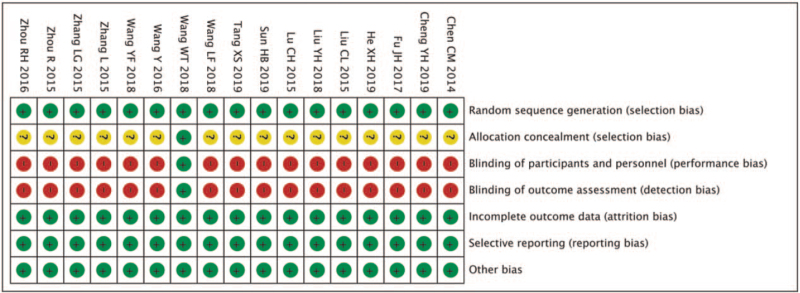
Included literature methodology assessment.

**Figure 3:**
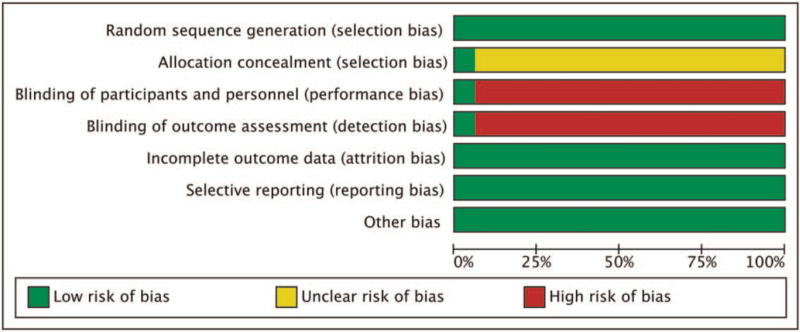
Statistical chart of evaluation entries.

The methodological evaluation of the included literature is shown in Figure 2. “+” in the figure indicates that low risk, “-” means that low risk, “?” indicates an unclear risk. One study^[16]^ was attributed 7 points, which is a low bias risk, and the rest^[5–15,17–21]^ scored 4 points, indicating moderate bias risk (Fig. 3).

### 3.4 Meta results analysis and discussion

#### 3.4.1 Perioperative outcomes

##### 3.4.1.1 Operating time

**Figure 4:**
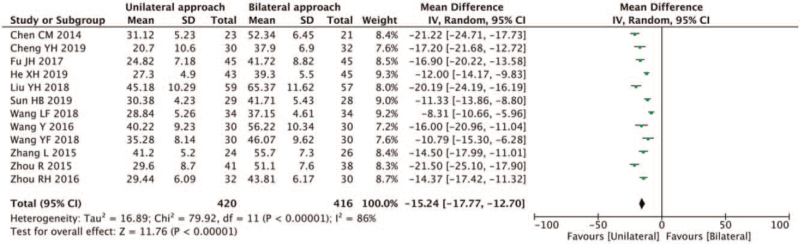
A forest plot for the operating time in unilateral and bilateral PVP groups. PVP = percutaneous vertebroplasty.

Sixteen studies^[5–17,19–21]^ reported operating time. Substantial heterogeneity was found in the included studies (*I*^2^ = 97% > 50%, and *Q* test *P* < .1). Based on the data of this study, it was highly suspected that the surgeons expertise caused the heterogeneity. Lu et al,^[5]^ Tang et al,^[8]^ Liu et al,^[14]^ and Wang et al^[16]^ were relatively heterogeneous. After excluding the above studies, significant heterogeneity was still present within the remaining 12 documents.^[6,7,9–13,15,17,19–21]^ A random-effects model was used for the meta-analysis (Fig. 4). The results showed that the operation time for unilateral PVP was less than for bilateral PVP (MD-15.24, 95%CI [−17.77,−12.70]; *P* < .05).

##### 3.4.1.2 X-ray exposure

**Figure 5:**
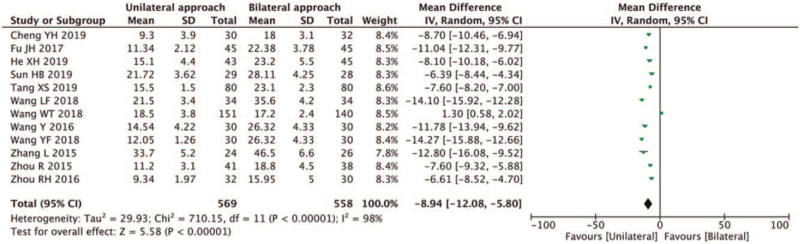
A forest plot for the X-ray exposure in unilateral and bilateral PVP groups. PVP = percutaneous vertebroplasty.

X-ray exposure was reported in 12 studies.^[6–8,10,12,13,15–17,19,21]^ High heterogeneity was found within the included studies (*I*^2^ = 98% > 50%, and the *Q* test *P* < .1). After sensitivity analysis, heterogeneity was still present, and a random-effects model was selected to perform the meta-analysis. The results showed that the radiation dose of patients was lower in the unilateral PVP group than in the bilateral PVP group (MD-8.94, 95% CI [−12.08, −5.80]; *P* < .01) (Fig. 5).

##### 3.4.1.3 Volume of injected cement

**Figure 6:**
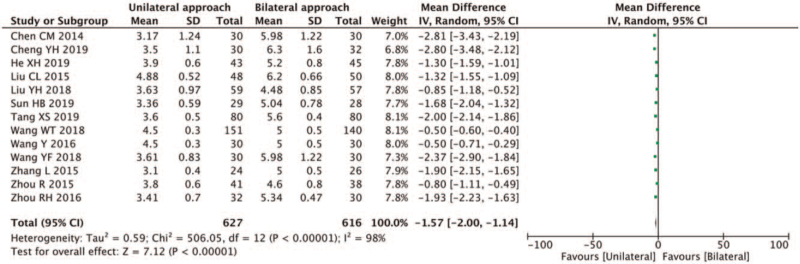
A forest plot for the volume of injected cement in unilateral and bilateral PVP groups. PVP = percutaneous vertebroplasty.

Thirteen studies reported the volume of injected cement.^[6–10,12,14,16,17,19–21]^ High heterogeneity was found within the selected studies (*I*^2^ = 98% > 50%, and the *Q* test *P* < .1). After sensitivity analysis, significant heterogeneity was still present. A random-effects model was selected for the meta-analysis. The results showed that the volume of injected cement in the unilateral PVP was less than that in the bilateral PVP (MD-1.57, 95% CI [−2.00, −1.14]; *P* < .05) (Fig. 6).

#### 3.4.2 Clinical outcomes

##### 3.4.2.1 Degree of vertebral height restoration

**Figure 7:**
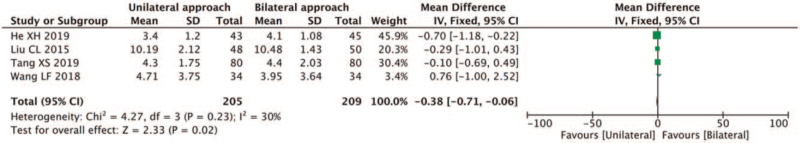
A forest plot for the degree of vertebral height restoration in unilateral and bilateral PVP groups. PVP = percutaneous vertebroplasty.

Six studies reported the degree of vertebral height recovery^[8,10,12–15]^; however, Wang et al^[10]^ only reported postoperative vertebral height, and Fu et al^[13]^ only reported the height recovery rate of the vertebral body; these 2 studies were excluded. A small heterogeneity was found in the remaining 4 studies^[8,12,14,15]^ (*I*^2^ = 30% <50%, and the *Q* test *P* = .23 > 0.1), and a fixed-effect model was selected for meta-analysis. The results showed that the postoperative height recovery after unilateral PVP was significantly less than after bilateral PVP (MD-0.38, 95% CI [−0.71, −0.06]; *P* = .02 < .05) (Fig. 7).

##### 3.4.2.2 Improvement of Cobb angle

**Figure 8:**
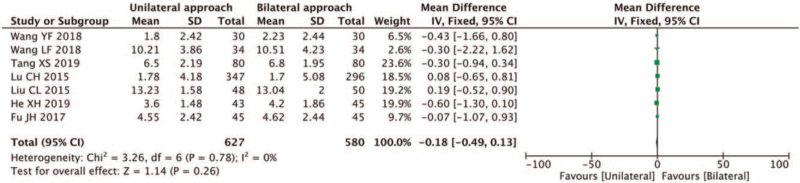
A forest plot for the improvement of Cobb angle in unilateral and bilateral PVP groups. PVP = percutaneous vertebroplasty.

Eight documents reported improvement of Cobb angle^[5,6,8,12–16]^; a strong heterogeneity was found in the selected studies (*I*^2^ = 86% > 50%, and the *Q* test *P* < .1). The sensitivity analysis showed that the study by Wang et al^[16]^ was different. The quality was relatively large, and a heterogeneous analysis was performed after removal. After excluding the study by Wang et al,^[16]^ lower heterogeneity (*Q* test *P* = .78, >.01) was found in the remaining seven articles^[5,6,8,12–15]^*I*^2^ = 0%; thus, a fixed-effect model was used for meta-analysis. The results showed that the postoperative recovery of Cobb angle in the unilateral puncture group was lower than that in the bilateral group (MD-0.18, 95% CI [−0.49, 0.13]; *P* = .26 > .01) (Fig. 8).

##### 3.4.2.3 VAS score

**Figure 9:**
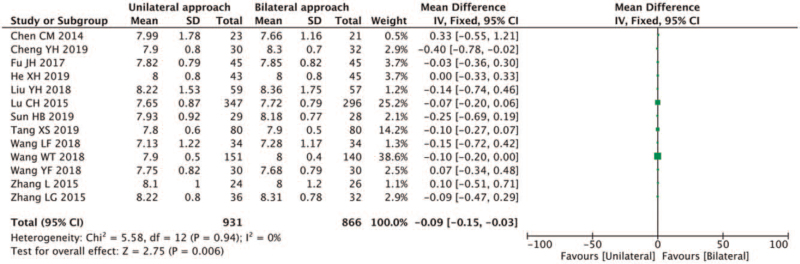
A forest plot for the VAS score in unilateral and bilateral PVP groups. PVP = percutaneous vertebroplasty.

VAS scores were reported in 13 studies^[5,6,8,9,12,13,15–21]^ and little heterogeneity was found in the preoperative data (*I*^2^ = 0%, *P* = .86 > .1). Accordingly, the pre-and postoperative data were merged and analyzed. Heterogeneity within the selected studies was relatively small (*I*^2^ = 0%, *P* = .94 > .1), and a fixed-effect model was selected for meta-analysis. The results showed that the VAS score at the last follow-up after unilateral PVP was lower than that after bilateral PVP (MD-0.09, 95% CI [−0.15, −0.03]; *P* = .006 < .05) (Fig. 9).

##### 3.4.2.4 Improvement of ODI score

**Figure 10:**
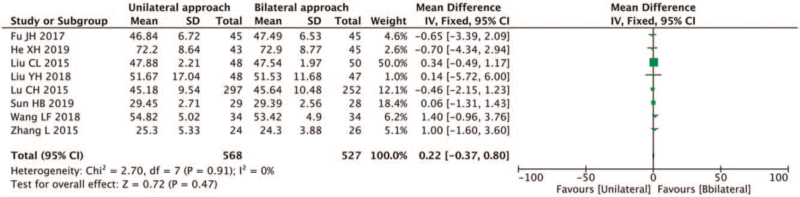
A forest plot for the improvement of ODI score in unilateral and bilateral PVP groups. PVP = percutaneous vertebroplasty.

Eleven studies reported on ODI scores,^[5,6,9,12–17,19,20]^ the study by Wang et al^[6]^ did not include follow-up data, and was excluded.^[9,12–17,19,20]^ Significant heterogeneity (*I*^2^ = 81% > 50%; *Q* test of *P* < .1) was found in the remaining ten studies.^[5]^ The sensitivity analysis showed that the study by Wang et al^[16]^ and Chen et al^[20]^ were different, and after the exclusion of these studies, heterogeneity analysis was carried out. Less heterogeneity was found in the remaining eight articles (*I*^2^ = 0%, and the *Q* test *P* = .910.01), and the fixed-effect model was used for meta-analysis. The results showed that the postoperative ODI improvement of the unilateral PVP was lower than that of the bilateral PVP (MD 0.22, 95% CI [−0.37, 0.80]; *P* = .47 > .05) (Fig. 10).

#### 3.4.3 Complications

##### 3.4.3.1 Cement leakage rate

**Figure 11:**
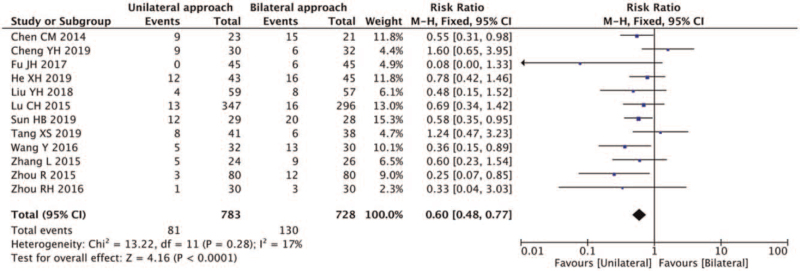
A forest plot for the cement leakage rate in unilateral and bilateral PVP groups. PVP = percutaneous vertebroplasty.

Cement leakage was reported in 15 studies,^[5,7–14,16–21]^ a strong heterogeneity was found in the selected studies (*I*^2^ = 54% > 50%, and the *Q* test *P* = .006 < .01). Furthermore, sensitivity analysis of the 15 studies found significant heterogeneity in studies by Liu et al,^[14]^ Wang et al,^[16]^ Zhang et al,^[18]^ and after excluding these 3 studies, heterogeneity analysis was carried out. The results showed relatively less heterogeneity in the remaining 12 articles (*I*^2^ = 17%, *Q* test *P* = .28, >.01). Thus a fixed-effect model was used for meta-analysis. Twelve studies^[5,7–13,17,19–21]^ showed that the risk of cement leakage of the unilateral puncture was lower than that of bilateral puncture (RR 0.6, 95% CI [0.48,0.77]; *P* < .01) (Fig. 11).

##### 3.4.3.2 Adjacent vertebral fracture

**Figure 12:**
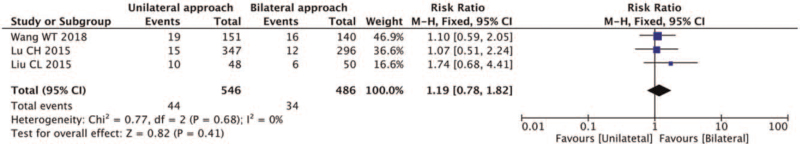
A forest plot for the adjacent vertebral fracture in unilateral and bilateral PVP groups. PVP = percutaneous vertebroplasty.

Three studies reported adjacent vertebral fracture.^[5,14,16]^ The heterogeneity between the selected studies in this study was small (*I*^2^ = 0%; *Q* test *P* = .68 > .01), and a fixed-effect model was selected for meta-analysis. The risk of adjacent vertebral fracture in unilateral PVP was higher than that in bilateral PVP, but the difference was not statistically significant (RR 1.19, 95% CI [0.78, 1.82]; *P* = .41 > .01) (Fig. 12).

##### 3.4.3.3 Neural root stimulation risk

**Figure 13:**
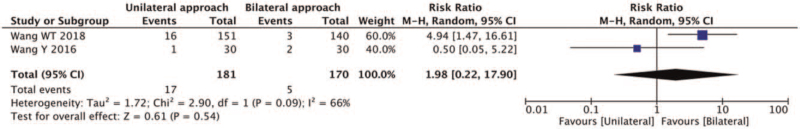
A forest plot for the neural root stimulation risk in unilateral and bilateral PVP groups. PVP = percutaneous vertebroplasty.

Two documents^[10,16]^ reported a neural root stimulation. Large heterogeneity (*I*^2^ = 66%; *Q* test *P* = .090) was found between these 2 studies, and a random-effect model was selected for meta-analysis. The risk of nerve stimulation in unilateral PVP was higher than that in bilateral PVP; however, the difference was not statistically significant (RR 1.98, 95% CI [0.22, 17.90]; *P* = .54 > .01) (Fig. 13).

#### 3.4.4 Bias analysis

**Figure 14:**
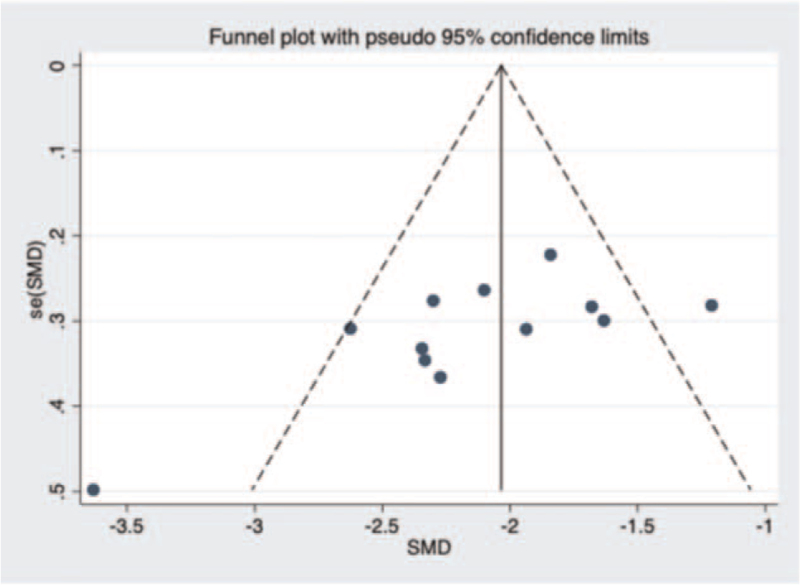
A funnel plot for the study about operation time in unilateral and bilateral PVP groups. PVP = percutaneous vertebroplasty.

**Figure 15:**
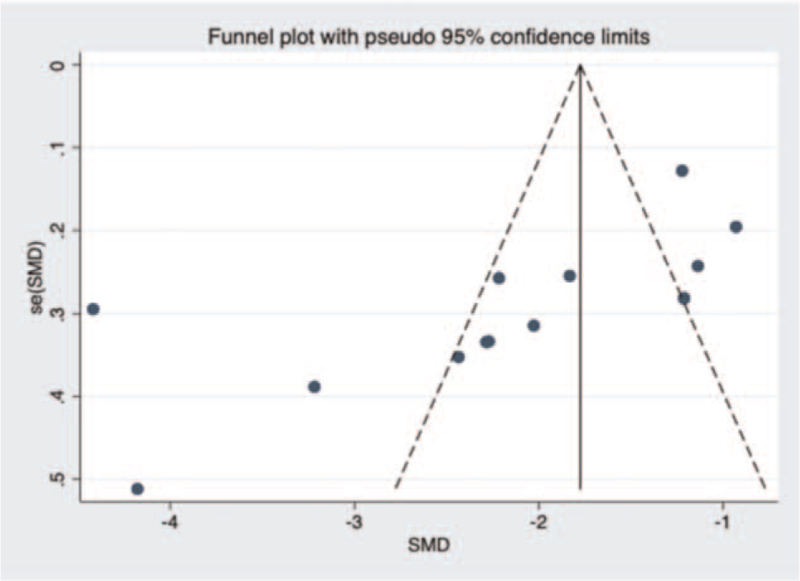
A funnel plot for the study about volume of injected cement in unilateral and bilateral PVP groups. PVP = percutaneous vertebroplasty.

**Figure 16:**
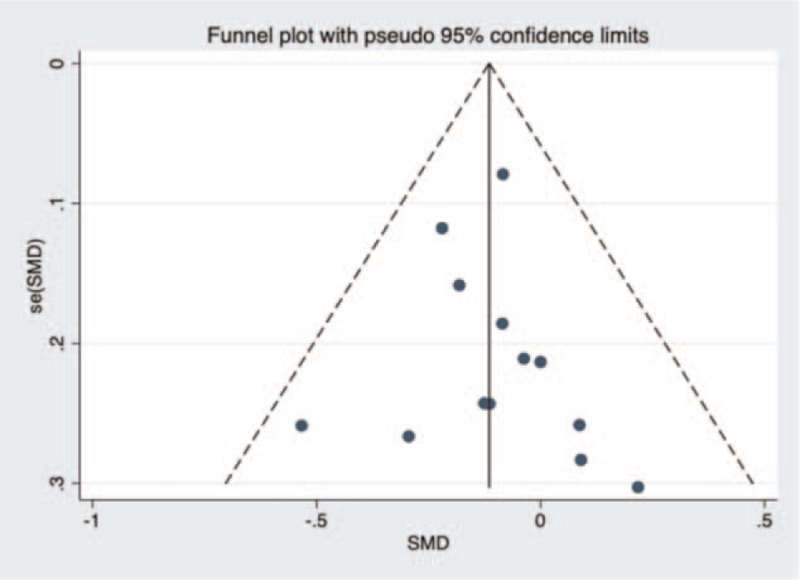
A funnel plot for the study about the VAS score in unilateral and bilateral PVP groups. PVP = percutaneous vertebroplasty.

**Figure 17:**
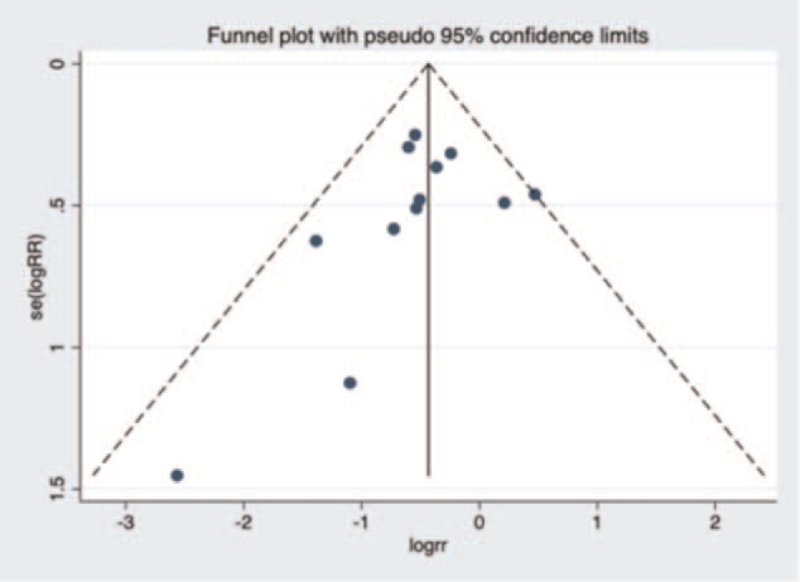
A funnel plot for the study about the cement leakage rate in unilateral and bilateral PVP groups. PVP = percutaneous vertebroplasty.

A funnel plot about operating time in unilateral and bilateral PVP groups was constructed (Fig. 14), and Begg test (*P* = .15 > .05) showed no evidence of publication bias in the 12 studies selected. Next, a funnel plot about volume of injected cement in unilateral and bilateral PVP groups was performed was (Fig. 15). After Begg (*P* = .001 < .05) and Egger test (*P* = .008 < .05), the publication bias of the 13 studies selected in this study was found to be significant. Then, a funnel plot about VAS score was constructed (Fig. 16). Begg (*P* = .3 > .05) and Egger test (*P* = .749 > .05) indicated that there was little possibility of publication bias in the 13 studies selected in this study. Finally, a funnel plot about cement leakage rate was constructed (Fig. 17), and Begg (*P* = .115 > .05), and Egger tests (*P* = .331 > .05) indicated that no publication bias in the 12 selected studies.

## 4 Discussion

Osteoporosis is a systemic disease presenting with reduced bone mass and microstructure damage and can degenerate into vertebral compression fractures.^[22,23]^ OVCFs can lead to chronic back pain, insomnia, reduced activity, depression, and even difficulty taking care of themselves in life.^[1]^ Percutaneous vertebroplasty is the most successful and effective minimally invasive surgical technique for OVCFs treatment at present.^[2]^ During this procedure, a bone substitute is injected into the injured vertebrae to reinforce it and help restore its biomechanical properties and morphology to a certain extent. Hypothetically speaking, more bone cement injections are usually associated with greater stability of the vertebral body and better pain relief. However, He et al^[24]^ reported that 3.8 mL of bone cement injection could relieve pain, and more bone cement would not help further relieve pain. Liebschner et al^[25]^ found that bone cement volume up to 3.5 cm^3^ or 14% of the L1 vertebral body volume helped restore the preoperative stiffness, and increasing the amount of bone cement injection could further increase the stiffness of the vertebral body. When the bone cement volume was greater than 4.9 cm^3^, the risk of adjacent vertebral fractures was increased.^[26]^ Jiang et al^[27]^ believed that a better degree of bone cement diffusion would lead to better postoperative symptom relief. Yan et al^[28]^ believed that bone cement injected during unilateral PKP is more dispersed and mainly distributed in the middle and front of the vertebral body, while for bilateral punctures, bone cement is mainly distributed on both sides. Accordingly, in the present study, cement injection in the unilateral puncture group was lower than in the bilateral puncture group (MD-1.57, 95% CI [−2.00, −1.14]; *P* < .05). Moreover, the VAS score at the last follow-up in our study was lower after unilateral puncture than after bilateral puncture (MD-0.09, 95% CI [−0.15, −0.03]; *P* = .006 < .05), which was inconsistent with findings of the previous studies.^[4,5]^ Furthermore, we found that the risk of fracture of adjacent vertebral bodies after both surgeries was significant (RR:1.19; 95%CI [0.78;1.82]; *Z* = 0.82, *P* = .41 > .01). And both approaches could improve postoperative function. It was found that the degree of improvement of ODI after unilateral PVP was higher than after bilateral PVP (MD 0.22, 95% CI [−0.37,0.80]; *P* = .47 > .05); however, the difference was not statistically significant (*Z* = 72, *P* > .05). To achieve a better degree of dispersion, unilateral PVP often requires a larger outer angle. Theoretically, unilateral PVP is more prone to nerve stimulation. Wang et al^[16]^ reported that the incidence of nerve root stimulation after unilateral PVP was higher than after bilateral punctures, while Wang et al^[10]^ reported the opposite observation. Herein, we found that higher incidence of nerve root stimulation associated to unilateral PVP than bilateral PVP, but the difference was not statistically significant (RR:1.98, 95%CI [0.22,17.90]; *P* = .54 > .01), which may be related to the small sample size and the low quality of included studies from the literature. More studies are required to substantiate our findings.

In a study where 1100 cases of vertebral plastic surgery were performed on 660 patients, 44% of patients suffered from bone cement leakage.^[29]^ According to Yeom et al,^[30]^ bone cement leakage can be classified into Types B, C, and S; type B leakage is bone cement leakage along the vertebral basement vein to the back edge of the vertebral body, type C leakage along with the vertebral body's bone cortex defect, and type S type leaks along the intervertebral vein. In our study, the incidence of cement leakage after unilateral puncture was lower than after bilateral puncture (RR:0.6, 95% CI [0.48, 0.77]; *Z* = 4.16, *P* < .01). Theoretically, unilateral PVP leakage presents predominantly as type B and bilateral PVP as type C. Unfortunately, the studies included in this meta-analysis did not analyze bone cement leakage types. Li et al^[31]^ reported that bone cement leakage was an independent risk factor for adjacent vertebral fractures, and Hansen et al^[32]^ believed that the higher the height restoration, the greater the risk of fractures in adjacent vertebral bodies. In our study, the postoperative height restoration was less (MD:0.38, 95% CI [−0.71, −0.06]; Z = 1.47, *P* = .02 < .05), and the postoperative Cobb angle restoration after unilateral PVP was lower than after bilateral PVP (MD−0.18, 95% CI [−0.49, 0.13]; *P* = .26 > .01). Given that the difference between the 2 groups was small and negligible, a significant risk of fracture of adjacent vertebral bodies after both surgeries was found (RR:1.19; 95% CI [0.78; 1.82]; *Z* = 0.82, *P* = .41 > .05).

Radiation exposure during X-ray exposure has deleterious effects on the human body. Excessive fluoroscopy can significantly increase the risk of cataracts, skin erythema, leukemia, thyroid cancer, and other malignant tumors.^[33]^ According to the International Commission on Radiological Protection guidelines, occupational exposure should be limited to an average maximum of 20 mSv/year over 5 years and should not exceed 50 mSv within a year.^[34]^ Indeed, doctors must minimize X-ray and radiation exposure. In our study, the X-ray exposure (MD-8.94, 95% CI [−12.08, −5.80]; *P* < .01) and operation times (MD-15.24, 95% CI [−17.77, −12.70]; *P* < .05) associated with unilateral PVP were less than bilateral PVP, and the difference was statistically significant.

It has been reported that unilateral PVP and bilateral PVP are effective surgical methods for OVCF; however, shorter operation and fluoroscopy duration and decreased cement injection were associated with unilateral PVP while achieving the same clinical effect as a bilateral puncture. Moreover, in the present study, unilateral PVP was not associated with increased incidence of cement leakage, nerve root stimulation, adjacent vertebral fractures, or other complications. Accordingly, unilateral PVP should be recommended when indicated.

However, some limitations still existed in this research.

First, the more indicators in both unilateral and bilateral groups could be analyzed, and it could be evaluated in the future. Second, the comparison among unipedicular approach, bilateral PVP and conservative therapy could be conducted in the future. Third, we did not perform a subgroup analysis to explore the potential sources of heterogeneity.

## Acknowledgments

We would like to thank our family members, and thanks for your support.

## Author contributions

**Conceptualization:** Yu Chen.

**Data curation:** Huang Zhang, Huihong Chen, Jinjun Zhang.

**Investigation:** Yu Chen, Huang Zhang, Huihong Chen, Zhiliang Ou.

**Methodology:** Huang Zhang, Yiping Fu.

**Project administration:** Yiping Fu.

**Resources:** Huang Zhang, Huihong Chen.

**Supervision:** Yu Chen.

**Validation:** Huang Zhang, Huihong Chen.

**Writing – original draft:** Yu Chen.

**Writing – review & editing:** Yu Chen.
